# High Macroalgal Cover and Low Coral Recruitment Undermines the Potential Resilience of the World's Southernmost Coral Reef Assemblages

**DOI:** 10.1371/journal.pone.0025824

**Published:** 2011-10-03

**Authors:** Andrew S. Hoey, Morgan S. Pratchett, Christopher Cvitanovic

**Affiliations:** 1 Red Sea Research Center, King Abdullah University of Science and Technology, Thuwal, Kingdom of Saudi Arabia; 2 ARC Centre of Excellence for Coral Reef Studies, James Cook University, Townsville, Queensland, Australia; Swansea University, United Kingdom

## Abstract

Coral reefs are under increasing pressure from anthropogenic and climate-induced stressors. The ability of reefs to reassemble and regenerate after disturbances (i.e., resilience) is largely dependent on the capacity of herbivores to prevent macroalgal expansion, and the replenishment of coral populations through larval recruitment. Currently there is a paucity of this information for higher latitude, subtropical reefs. To assess the potential resilience of the benthic reef assemblages of Lord Howe Island (31°32′S, 159°04′E), the worlds' southernmost coral reef, we quantified the benthic composition, densities of juvenile corals (as a proxy for coral recruitment), and herbivorous fish communities. Despite some variation among habitats and sites, benthic communities were dominated by live scleractinian corals (mean cover 37.4%) and fleshy macroalgae (20.9%). Live coral cover was higher than in most other subtropical reefs and directly comparable to lower latitude tropical reefs. Juvenile coral densities (0.8 ind.m^−2^), however, were 5–200 times lower than those reported for tropical reefs. Overall, macroalgal cover was negatively related to the cover of live coral and the density of juvenile corals, but displayed no relationship with herbivorous fish biomass. The biomass of herbivorous fishes was relatively low (204 kg.ha^−1^), and in marked contrast to tropical reefs was dominated by macroalgal browsing species (84.1%) with relatively few grazing species. Despite their extremely low biomass, grazing fishes were positively related to both the density of juvenile corals and the cover of bare substrata, suggesting that they may enhance the recruitment of corals through the provision of suitable settlement sites. Although Lord Howe Islands' reefs are currently coral-dominated, the high macroalgal cover, coupled with limited coral recruitment and low coral growth rates suggest these reefs may be extremely susceptible to future disturbances.

## Introduction

Coral reefs, and particularly reef-building corals, are subject to a diversity of disturbances, ranging from very localised events (e.g. discrete predation events) that kill or injure individual coral polyps, to ocean-scale bleaching events associated with climate change [1], [2]. Moreover, the diversity, frequency and severity of disturbances affecting reef corals are increasing, especially those disturbances associated with climate change [3], [4]. Consequently, the long-term persistence of coral populations will depend upon their resilience [5], [6], which is affected by i) resistance, which is the ability of established corals to withstand different disturbances, and/or ii) recovery, which is the rate at which new coral colonies recruit, survive and grow to colonise available space and/or the regrowth of remnant coral tissues following declines in population size [1], [7].

Resilience, and especially recovery, of coral populations is strongly influenced by interactions between corals and macroalgae. Excessive growth and coverage of macroalgae may reduce the growth, survivorship, and fecundity of established coral colonies [8]–[11]. Macroalgae may also limit coral recruitment and the recovery potential of reefs by inhibiting settlement [12] and smothering new coral recruits [10]. Given the potential importance of macroalgae in the functioning and resilience of coral reef ecosystems, it is not surprising that considerable research effort has focused on determining the factors that influence macroalgal distributions. Collectively, these studies have identified a number of physical and biological mechanisms that may shape coral reef algal communities; including herbivory, eutrophication, hydrodynamics and sedimentation (e.g., [13], [14]). Of these mechanisms, herbivory is widely accepted as a key determinant of benthic community succession and algal community structure on coral reefs (reviewed by [15]).

On reefs with intact fish assemblages, in excess of ninety percent of the net daily production of the algal community is consumed by herbivores [16]–[18]. At reduced levels of herbivory, the balance between the production and consumption of algae is disrupted. Several studies have demonstrated that the exclusion of herbivorous fishes from small areas of reef leads to a shift from a low biomass of highly productive algal turfs and grazing resistant crustose coralline algae (CCA) to a high biomass of less productive fleshy macroalgae (e.g., [10]). Over larger scales, regional reductions in herbivores through overfishing and the subsequent degradation of these reefs to macroalgal dominance has highlighted the importance of herbivores in structuring benthic communities and maintaining a healthy balance between corals and macroalgae [19], [20].

Within tropical reef systems, variation in the abundance and community structure of herbivorous fishes has often been cited as a significant factor influencing algal communities. Strong negative relationships between herbivore biomass and the cover of fleshy macroalgae have been documented for the Great Barrier Reef (GBR) [21], [22], Caribbean [23], [24], and Hawaiian [25] reef systems. While these relationships appear well established for tropical coral reefs, such associations are yet to be examined for marginal subtropical reefs. High cover of fleshy macroalgae, while often viewed as a sign of degradation on tropical coral reefs (but see [22], [26]), appears to be a relatively ‘natural’ state on subtropical reefs [27], [28]. Quantifying the relationships between benthic composition and herbivore community structure will improve our understanding of the processes that structure these high latitude reefs.

Subtropical reefs lie on the latitudinal limit for coral reef growth [29], and support a unique diversity of tropical and temperate taxa [30], [31]. To date, subtropical reefs have largely escaped the extreme effects of increasing seawater temperatures that have impacted tropical reefs globally [32], [33]. This apparent stability, coupled with evidence of climate-induced poleward shifts of coral reef taxa over both geological [5], [34] and ecological time scales [35]–[37] has led to suggestions that these subtropical reefs may perform an important role as refugia from the impacts of climate change. While not exposed to the frequency or intensity of events affecting tropical reefs, subtropical reefs are nonetheless subject to a range of disturbances, including coral bleaching, disease, and crown-of-thorns starfish outbreaks (e.g., [32], [38]–[40]). The susceptibility of subtropical reefs to climate change may depend on their regenerative capacity following these relatively infrequent disturbances, rather than their ability to resist multiple extreme events.

The objective of this study was to assess the potential resilience of benthic reef assemblages at Lord Howe Island, southern Australia. The specific aims of this study were to i) provide a comprehensive assessment of the benthic community structure of Lord Howe Island, ii) quantify the herbivorous fish communities in order to examine the relationship between herbivory and benthic composition, and iii) quantify rates of coral recruitment. This study will facilitate predictions about the likely recovery and resilience of these coral reef habitats following episodic disturbances associated with climate change or other anthropogenic stresses. This is a critical and timely goal given the stressors to which coral reefs are currently being exposed.

## Methods

### Ethics Statement

The activities for this study were conducted under permission from the New South Wales Marine Park Authority (Permit Number LHIMP/R/2010/004). Only visual censuses of fish and benthic communities were conducted during this study; no fauna or flora were collected or manipulated.

### Study Sites

**Figure 1:**
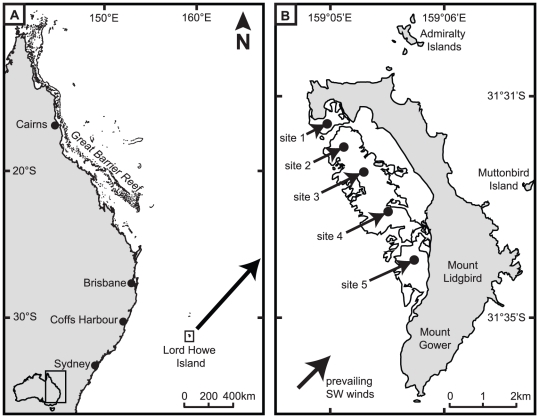
Map showing location of study sites. **A.** Map of the east coast of Australia showing the geographic location of Lord Howe Island. **B.** Map of Lord Howe Island, showing the location of the five sites used to quantify the benthic composition and herbivorous fish community. At each site the three habitats were sampled; the reef slope (8–10 m depth), the reef crest (2–4 m), and the back reef (1–3 m).

This study was conducted in April-May 2010 at Lord Howe Island (31°32′S, 159°04′E), 630 km east of mainland Australia (Figure 1a). Surveys of the benthic composition and fish community structure were conducted at 5 sites evenly spaced along the length of extensive reef that mostly encloses a lagoon on the western side of the island (Figure 1b). At each site, surveys were conducted in three distinct habitats; i) the reef slope, ii) reef crest, and iii) shallow back reef. The only exception was the reef crest habitat at site 1. Extremely unfavourable weather on the final days of the study precluded access to this area and consequently no surveys were performed. The reef slope and crest were directly exposed to the prevailing south-west trade winds. The reef slope was at a depth of 8–10 m on the steeply inclined region of the reef. The reef crest (2–4 m depth) was the region that marked the transition between the steeply inclined reef slope and the extensive shallow region of the reef. The back reef was at the leeward margin of the reef flat at a depth of 1–3 m and marked the transition from the reef flat to deeper lagoonal habitats dominated by sand.

### Benthic Composition

The benthic composition of reef habitats was documented using 50-m point-intercept transects, following Pratchett et al. [41]. Six replicate transects were conducted within each habitat at each of the five sites. A total of 50 points were surveyed on each transect, spaced at 1-m intervals. Any scleractinian (hard) corals, alcyonacean (soft) corals, or macroalgae (> 5 mm in height) underlying each survey point were identified to genus. For survey points that did not intersect live coral or macroalgae, the underlying habitat was categorised as algal turf or epilithic algal matrix (EAM; <5 mm in height), CCA, rubble, or sand.

### Juvenile Corals

Juvenile corals were defined as any corals that were <50 mm maximum diameter and visible with the naked eye, following Rylaarsdam [42]. However, corals <10 mm diameter are generally very cryptic and difficult to detect without specialist equipment [43]; in this study the smallest corals detected were 10 mm diameter. It was our intention to use densities of juvenile corals as a proxy for recruitment by sexually derived larvae, and therefore only attached colonies were counted. Care was also taken to exclude small corals that were formerly part of a larger colony (remnants or fragments). Densities of juvenile corals were quantified using replicate 10×1 m belt transects, whereby the diver (MSP) moved slowly (<1 m.minute^−1^) along the transect searching for juvenile corals within and under all substrata, following Pratchett et al. [41]. Any juvenile corals detected were classified to genus. Six replicate transects, coinciding with the start of the benthic transects (described above) were sampled within each habitat at each site.

### Herbivorous Fishes

Species-level surveys of all roving herbivorous fishes were conducted using a underwater visual census along belt transects. One diver (ASH) recorded all nominally herbivorous fishes from the families Acanthuridae (surgeonfishes), Aplodactylidae (marblefishes), Labridae (parrotfishes) and Kyphosidae (drummers) greater than 10 cm total length (TL) within a 5 m wide belt that extended from the reef substratum to the surface of the water. A second diver swam behind the first deploying a 50 m transect tape. This procedure minimised disturbance prior to censusing and allowed a specified area to be censused. Individual fishes were identified to species and placed into 5 cm size categories. Care was taken not to re-census fish that left and subsequently re-entered the transect area. The fish surveys were conducted along the same 50-m transects used for the benthic surveys, with six replicate transects being conducted within each habitat at each site. Fish densities were converted to biomass using published length-weight relationships for each species, following Hoey and Bellwood [44].

Herbivorous fishes were categorised as either macroalgal browsers or grazers (including scraping and excavating parrotfishes) based on the algal material they target [45]–[49]. Specifically, *Leptoscarus vaigiensis*, *Naso annulatus*, *Naso unicornis*, *Prionurus maculatus*, *Girella cyanea*, *Kyphosus* spp., and *Crinodus lophodon* were identified as macroalgal browsers. The remaining species were considered grazers as they typically feed on the EAM and/or CCA and are not likely to consume larger macroalgae. This functional dichotomy, while not mutually exclusive, is useful as it highlights the distinction between those species that have the capacity to prevent (i.e., grazers) or potentially reverse (i.e., browsers) shifts to macroalgal-dominance on coral reefs [49], [50].

### Statistical analyses

Variation in the cover of live coral, macroalgae, EAM and CCA, the density of juvenile corals, and the biomass of herbivorous fishes was compared among sites and habitats using a series of two-factor ANOVAs, with habitat and site considered fixed factors. The low cover of soft coral, rubble and sand precluded any meaningful comparisons for these benthic categories. Type IV sums of squares were used to adjust for the lack of data for the reef crest at site 1. Assumptions of the ANOVA were examined by residual analysis. Subsequently all substratum categories were arcsin-square root transformed, the density of juvenile corals was log transformed, and the biomass of herbivorous fishes was square-root transformed. Student-Newman-Keuls (SNK) multiple comparisons were used to identify which means contributed to any significant differences detected. Relationships between benthic categories, the density of juvenile corals, and herbivore biomass were examined using Pearsons correlation coefficient.

Principal component analyses (PCA) were used to investigate variation in the benthic community composition and herbivorous fish assemblages among sites and habitats. The analyses were based on the covariance matrix of the mean proportion of each substratum category, and the mean biomass of each herbivorous species in each habitat within each site, respectively. The biomass of each herbivorous species was square-root transformed and uncommon species (<1 individual per transect) were pooled to higher taxonomic levels.

## Results

### Benthic composition

**Figure 2:**
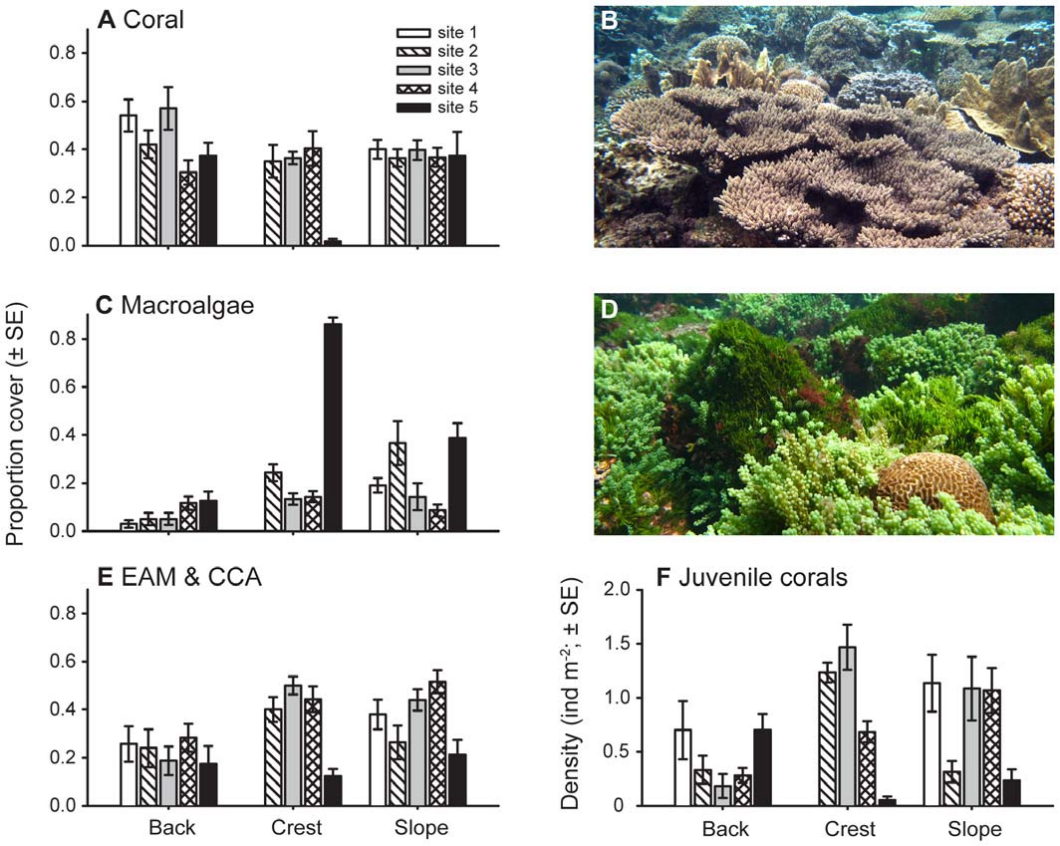
Spatial variation in benthic assemblages on Lord Howe Island. Variation in the (**A**) cover of live scleractinian coral, (**C**) cover of macroalgae, (**E**) cover of the crustose coralline algae (CCA) and epilithic algal matrix (EAM), and (**F**) density of juvenile corals (<50 mm diameter) among three habitats and five sites on Lord Howe Island. Each mean is based on six transects. (**B**) A diverse coral-dominated assemblage in the back reef at site 3 on Lord Howe Island, (photo A.H. Baird) and (**D**) a macroalgal-dominated assemblage on the reef crest at the site 5, the southernmost site. High cover of *Caulerpa racemosa* and *C. taxifolia* surrounding small faviid colony.

Overall, mean coral cover was 37.4% (±1.9 SE) at Lord Howe Island, but displayed significant variation among habitats and sites (habitat×site: F_7,70_ = 5.59, p <0.001; Figure 2a–d). Coral cover was mostly similar among habitats and sites, except on the reef crest of the southernmost site (i.e., site 5), where coral cover was <2% (Figure 2a). The cover of macroalgae (overall mean = 20.9±2.5%) was generally lower than that of coral, but displayed higher variability among habitats and sites (habitat×site: F_7,70_ = 10.52, p<0.001; Figure 2c). Macroalgal cover ranged from 13.3–86.0% and 8.7–38.7% on the reef crest and slope, respectively, with the greatest cover being recorded at site 5 for both habitats (Figure 2c,d). In contrast, macroalgal cover was generally lower and displayed limited variation among sites in the back reef (3.0–12.7%). Details of the taxonomic composition of the macroalgal assemblage are given in the supplementary material (Table S1). The cover of CCA and EAM, collectively, displayed significant variation among habitats (F_2,70_ = 7.85, p<0.001) and sites (F_4,70_ = 7.48, p<0.001) with the cover being lowest on the back reef and at site 5 (Figure 2e). Further details of the ANOVA's and SNK multiple comparisons are given in the supplementary material (Table S2 and S3).

**Figure 3:**
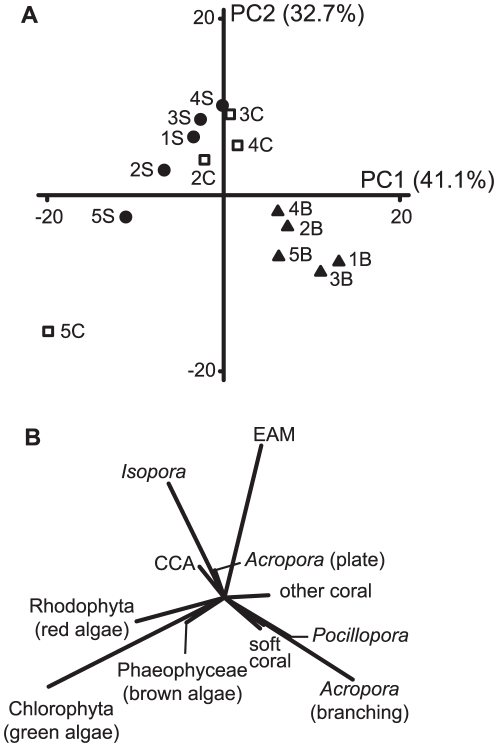
Principal component analysis showing the relationships among benthic assemblages on Lord Howe Island. (**A**) Ordination plot showing the relationship between fourteen locations. Each location is based on six 50-m point-intercept transects. Solid circles: reef slope locations; open squares: reef crest locations; solid triangles: back reef locations. Numbers refer to sites identified in Figure 1. (**B**) Substratum category loadings showing the relative contributions of each substratum to the observed differences in benthic community structure. CCA: crustose coralline algae; EAM: epilithic algal matrix.

The taxonomic composition of the benthic assemblages also varied among sites and habitats. The PCA showed clear among-habitat differences in the benthic community structure, with the first two axes explaining 73.8% of the total variation (Figure 3a). The back reef habitats were clearly separated from the reef crest and slope habitats along the first principal component and were characterised by a high cover of branching *Acropora* and *Pocillopora*. The benthic structure of the reef crest and slope displayed greater similarity among sites than habitats, with the majority of sites being characterised by a high cover of *Isopora* and EAM (Figure 3b). The only exception to this was the southernmost reef crest and slope (i.e. site 5) that had an extremely high cover of macroalgae dominated by *Caulerpa* (Chlorophyta), which covered 42.7 and 30.7% of the substratum on the reef crest and slope, respectively.

### Juvenile corals

The densities of juvenile corals (<5 cm diameter) recorded at Lord Howe Island were highly variable, ranging from 0 to 22 individuals per 10 m^2^ (mean = 7.7 ± 0.8 SE ind.10 m^−2^), and was influenced by an interaction between habitat and site (F_7,70_ = 11.67, p<0.001; Figure 2f). The density of juvenile corals was generally lower on the back reef than on the reef crest, with the highest densities being recorded on the reef crest of sites 2 and 3 (12.3–14.7ind.10 m^−2^; Figure 2f). The only exception to this was at site 5 where very few juvenile corals were recorded on the reef crest (0.5 ± 0.3ind.10 m^−2^). The reef crest at this location was covered with macroalgae (mean cover = 86.0%). The juvenile coral assemblage was dominated by *Isopora* (38%), *Pocillopora* (28%), and *Porites* (28%) on the reef slope; *Acropora* (30%), *Isopora* (19%), and *Pocillopora* (13%) on the reef crest; and *Isopora* (24%), *Pocillopora* (20%), and *Seriatopora* (17%) in the back reef.

### Herbivorous fish communities

**Figure 4:**
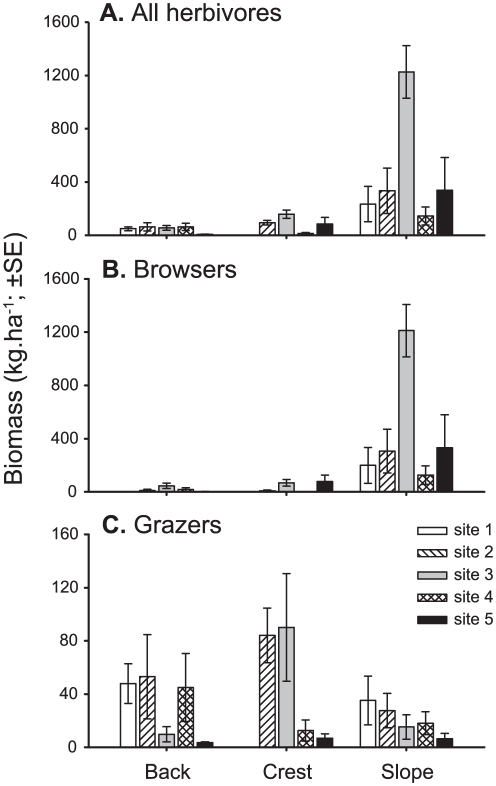
Spatial variation in herbivorous fish biomass on Lord Howe Island. Variation in (**A**) all roving herbivorous fishes, (**B**) macroalgal browsing fishes, and (**C**) grazing fishes among three habitats and five sites on Lord Howe Island. Each mean is based on six 50-m belt transects. Note the difference in the y-axis scales for the two functional groups.

Macroalgal browsing species dominated the herbivorous fish community on Lord Howe Island, accounting for 84.1% of the total herbivore biomass, as opposed to only 15.9% for grazing taxa (scraping parrotfishes: 14.0%; excavating parrotfishes: 0.7%; algal croppers: 1.2%). Total herbivorous fish biomass varied among habitats (F_2,70_ = 17.09, p<0.001) and sites (F_4,70_ = 7.01, p<0.001), with the biomass being greatest on the reef slope and at site 3 (Figure 4a). Similarly, the biomass of browsing fishes was greatest on the reef slope across all sites (F_2,70_ = 36.70; p<0.001), with site 3 having the greatest biomass across all habitats (F_4,70_ = 8.20; p<0.001; Figure 4b). In contrast, the biomass of grazing fishes decreased significantly from the northern sites (i.e. sites 1 and 2) to the southernmost site (F_4,70_ = 3.50; p = 0.012; Figure 4c), but displayed no significant variation among habitats (F_2,70_ = 1.58; p = 0.212).

**Figure 5:**
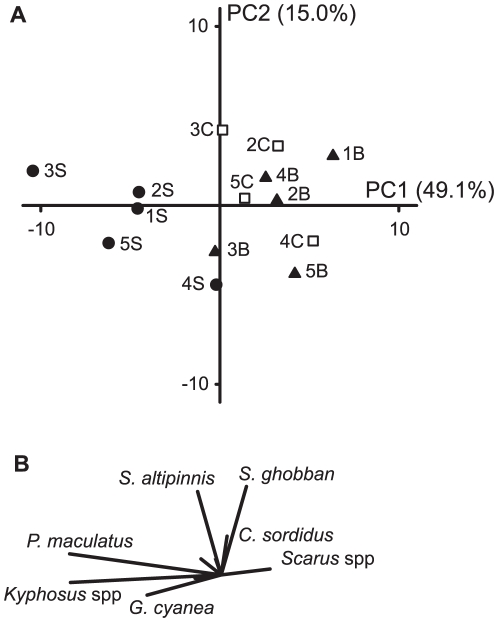
Principal component analysis showing the relationships among herbivorous fish assemblages on Lord Howe Island. (**A**) Ordination plot showing the relationship between fourteen locations. Each location is based on six 50-m belt transects. Symbols as for Figure 3. (**B**) Species loadings showing the relative contributions of each species to the observed differences in herbivorous fish community structure. Species names: *Girella cyanea* (Kyphosidae); *Prionurus maculatus* (Acanthuridae); *Chlorurus sordidus*, *Scarus altipinnis*, *Scarus ghobban* (Labridae).

The taxonomic composition of the herbivorous fish community displayed clear among-habitat differences with the first two axes of the PCA explaining 64.1% of the total variation (Figure 5a). The reef slope was clearly separated from all reef crest and back reef sites along the first principal component and was characterised by a high biomass of the macroalgal browsing fishes, *Kyphosus* spp. (primarily *Kyphosus bigibbus*), *Prionurus maculatus*, and *Girella cyanea* (Figure 5b). In contrast, the reef crest and back reef sites were characterised by grazing taxa, namely the scraping parrotfishes, *Scarus ghobban* and *Scarus altipinnis*, and to a lesser extent the excavating parrotfish, *Chlorurus sordidus* (Figure 5b).

### Relationships among variables

**Figure 6:**
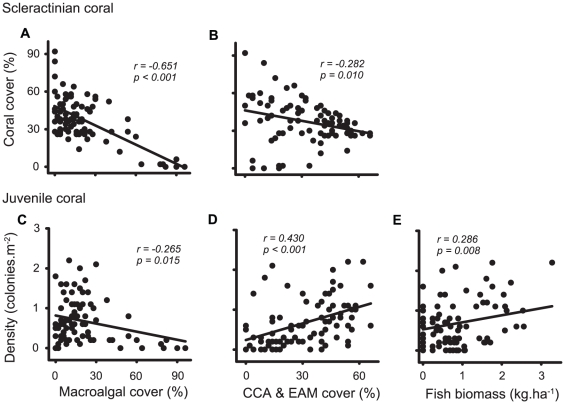
Relationships among major benthic categories and the biomass of grazing fishes on Lord Howe Island. Correlations between the cover of live coral and (**A**) the cover of macroalgae, and (**B**) the cover of EAM and CCA. Correlations between the density of juvenile corals and (**C**) the cover of macroalgae, (**D**) the cover of EAM and CCA, and (**E**) the biomass of grazing fishes. Each point represents values from individual transects (n = 84). The best-fit relationships (linear) are given as solid lines, along with *r* and *p* values.

The cover of live scleractinian coral was negatively related to the cover of macroalgae, and the cover of CCA and EAM (Table 1; Figure 6a,b). Similarly, the density of juvenile corals was negatively related to macroalgal cover (Figure 6c), but displayed a positive relationship to the cover of CCA and EAM (Figure 6d). There was no significant relationship between the live coral cover and the density of juvenile corals (Table 1). Overall, herbivorous fish biomass (either collectively or browsing and grazing taxa independently) was a poor predictor of benthic communities, and displayed no relationship to the cover of macroalgae or live coral (Table 1). The only exception to this was the biomass of grazing fishes, which was positively related to both the density of juvenile corals (Figure 6e) and the cover of CCA and EAM (Table 1).

**Table 1:** Relationships between benthic variables and herbivorous fish biomass on Lord Howe Island.

	Macroalgae	Live coral	CCA and EAM	Juvenile coral
Live coral	−0.651***			
CCA and EAM	−0.301**	−0.2816	**	
Juvenile coral	−0.265*	0.164 ns	0.430***	
Browsing fish biomass	0.031 ns	0.049 ns	0.063 ns	0.131 ns
Grazing fish biomass	−0.147 ns	−0.006 ns	0.222*	0.286**
Total herbivore biomass	0.012 ns	0.049 ns	0.092 ns	0.168 ns

Correlations between the cover of the major benthic taxa, density of juveniles corals, and the biomass of herbivorous fishes on Lord Howe Island. Analyses were based on 85 paired transects. Pearsons correlation coefficient is shown.

*<0.05.

**<0.01.

***<0.001; ns non-significant.

## Discussion

Subtropical reefs, located at the latitudinal limit of reef formation, are typically characterised by relatively low coral cover and high abundance of fleshy macroalgae [27]. This study revealed that the coral cover at Lord Howe Island, the worlds' southernmost coral reef, was higher (overall mean = 37.4%) than most other subtropical reefs (ca. 3.9–25.3%; [27], [28], [51], [52]), and directly comparable to lower latitude reefs of the GBR, where mean coral cover typically ranges from 18.3–27.0% on inshore reefs to 30.7–33.6% on offshore reefs [22], [53]. Our estimates of coral cover are broadly comparable to those previously recorded from Lord Howe Island (ca. 25–44%; [26], [30], [31], [54]), suggesting that coral cover has changed little over past three decades. This apparent stability of Lord Howe Islands' coral communities may imply they are relatively resilient, however, these reefs have largely escaped the stressors (i.e. bleaching and disease) that have caused marked declines in coral cover (ca. > 10%) on tropical Indo-Pacific reefs over the same period [1]. A recent bleaching event highlights that these subtropical reefs are not immune, with extensive bleaching (up to 90% of colonies) and some bleaching-induced mortality (up to 25% of colonies) in localised areas of the Lord Howe Island lagoon [40]. Our results suggest that the high macroalgal cover, low recruitment of juvenile corals, and low biomass of herbivorous fishes may limit the capacity of these reefs to recover from such disturbances.

Densities of juvenile corals in the present study (mean = 0.8 ind.m^−2^) were markedly lower than estimates from tropical reefs using similar methods (ca. 4–80 ind.m^−2^: [41], [55], [56]). This is in marked contrast with Harriott [38], [57], who reported rates of coral recruitment to artificial surfaces (i.e., potential recruitment) within the Lord Howe Island lagoon to be directly comparable to those of the GBR. This variation may reflect differences in the availability of suitable settlement sites among studies, or differences in post-settlement mortality among latitudes. The cover of live coral and the density of juvenile corals among locations were both negatively correlated with fleshy macroalgae in the present study. In particular, transects with very high (> 60%) cover of macroalgae, had almost no adult or juvenile corals. This suggests that high cover of macroalgae limits establishment and/or persistence of scleractinian corals, and is supported by experimental studies that have demonstrated that excessive growth of macroalgae inhibits growth, survivorship, fecundity and/ or recruitment of scleractinian corals (e.g., [10], [11]). On tropical coral reefs, phase-shifts from coral- to macroalgal-dominated reef scapes are often precipitated by an acute disturbance event (e.g., cyclones or bleaching) that causes extensive coral loss (e.g., [19]). There are however, some instances where sustained increases in cover and biomass of macroalgae eventually overwhelm scleractinian corals, leading to gradual shifts in the dominant biota [58]. In either case, reversing these phase-shifts on tropical reefs requires an increased abundance of specific herbivores that will feed on macroalgae and ultimately clear space for new coral recruits [50], [59]. At Lord Howe Island, however, it is unclear whether there were ever sufficient herbivorous fishes to effectively regulate macroalgal abundance, or if these areas of high macroalgal cover are a natural state on this, and other, subtropical reefs. The need for intervention will only become apparent by assessing temporal patterns in coral-algal dynamics (e.g., are macroalgae increasing in abundance within formerly coral-dominated habitats?).

Aside from impacts on coral reef resilience, limitations to recruitment by scleractinian corals in sub-tropical locations will limit the capacity for poleward shifts in the distribution of corals, and therefore many other reef-associated organisms. Given increasing temperatures and specific thermal tolerances of many tropical species, sub-tropical reefs are considered to be potentially important refuges for climate sensitive reef species [34], [60]. Accordingly, the number of coral species recorded at Lord Howe Island increased from 64 in 1979 to 83 in 1993 [30]. This increase is presumably due to colonisation by larvae spawned on the GBR, but may also reflect differences in sampling locations or the taxonomic status of species among studies. Even so, Harriott [57] suggested that there is limited capacity for GBR corals to successfully disperse to Lord Howe Island, due to limited planktonic phases for most species. Given recent improvements in understanding of connectivity among coral reef populations, and capacity for long-disturbance dispersal [61], the likelihood of connections between the GBR and Lord Howe Island, may need to be revisited.

Macroalgal cover showed no significant relationships with the biomass of herbivorous fishes, either collectively or the two functional groups (i.e. grazers and browsers), independently. Similarly, Vroom and Braun [28] found no relationship between macroalgal cover and the densities of herbivorous fishes or urchins on the subtropical reefs of the Northwestern Hawai'ian Islands (NWHI). Although the distribution of herbivorous urchins were not quantified, previous studies at Lord Howe Island have shown that urchin densities tend to be greatest in the deeper reef slope habitats and lowest in the lagoon or back reef (e.g., [31], [62]). Therefore, it appears unlikely that grazing by herbivorous urchins could explain the observed variation in macroalgal cover.

Overall, the biomass of herbivorous fishes on Lord Howe Island (mean = 204 kg.ha^−1^) was considerably lower than that of tropical reefs (ca. 400–3000 kg.ha^−1^; [22], [26], [44], [46], [63]). Although the higher macroalgal cover typical of subtropical reefs may be related to the lower densities or biomass of herbivorous fishes on these reefs [64], [65], it appears that herbivory alone cannot explain among-habitat variation in macroalgal communities on Lord Howe Island. The exceptionally high macroalgal cover at site 5, our southernmost site, (reef crest = 86.0%; reef slope = 38.7%) may be related to other factors such as variation in wave action, or its proximity to Mt Lidgbird and Mt Gower (777 and 875 m in height, respectively) which may reduce light availability, alter wind forces, or increase freshwater input. While wave energy has been shown to influence the morphology and distribution of macroalgae on some reefs (e.g., [13]), all sites in the present study were oriented to the prevailing south-west swells and likely to experience similar wave energy.

The functional composition of Lord Howe Islands' herbivorous fish community differed markedly from those of tropical low-latitude reefs. The herbivorous fish community of Lord Howe Island was dominated by macroalgal browsing species (84.1%) with relatively few grazing taxa. In marked contrast, grazing fishes typically account for over 80% of the total herbivorous fish biomass on tropical reefs (e.g., [22], [63], [66], [67]), and up to 60% of the total herbivore biomass on subtropical NWHI reefs [66]. Even on Elizabeth and Middleton Reefs, 200–260 km north of Lord Howe Island, large excavating and scraping parrotfishes (*Chlorurus frontalis*, *C. microrhinos*, and *S. altipinnis*) are abundant on exposed reef crests [52]. This shift in functional composition represents a 10- to 70-fold reduction in the biomass of grazing fishes on Lord Howe Island. Grazing fishes typically feed on the EAM (primarily filamentous algae, macroalgal propagules, and detritus) and CCA, and by removing macroalgal propagules not only create suitable settlement sites for coral larvae [24] but also play an important role in helping reefs to resist shifts to alternate states and reassemble following disturbances [1], [10]. Despite their low biomass, grazing fishes were positively related to both the density of juvenile corals and the cover of bare space (i.e., CCA and EAM) in the present study. While far from conclusive, these results suggest that grazing fishes on Lord Howe Island may enhance realised recruitment of corals through the provision of suitable settlement sites.

Coral reefs are under increasing pressure from direct anthropogenic disturbances and climate change, with projected increases in the severity and frequency of disturbances likely to cause accelerated declines in coral cover and structural complexity of reef habitats [3], [4]. The ability of coral communities to reassemble and regenerate after disturbances is critical to their long-term persistence, and is dependent on both the ongoing replenishment of coral populations through larval recruitment, as well as the maintenance of suitable substrates for coral settlement and growth [10]. In this respect, herbivores that limit macroalgal expansion and overgrowth of reef substrata are critical to reef resilience [1]. The results of this study suggest, that despite the benthic communities of Lord Howe Island being dominated by live scleractinian coral, the high macroalgal cover, coupled with the low level of coral recruitment (as proxied by juvenile coral density) and coral growth [68] may limit the capacity of this reef to reassemble following disturbances. Elucidating the mechanisms that regulate macroalgal abundance at Lord Howe Island and other subtropical reefs may further our understanding of the potential for phase-shifts on coral reefs throughout the world.

## Supporting Information

Table S1 **Spatial variation in macroalgal assemblages of Lord Howe Island.** Summary of the composition of macroalgal assemblages across three habitats within each of five sites on Lord Howe Island. Mean percentage cover of each of the major macroalgal taxa (based on six 50-m transects) are given.
(DOCX)

Table S2 **Summary of 2-way ANOVA's comparing benthic and fish communities among habitats and sites on Lord Howe Island.** Variation in (**A**) cover of live scleractinian coral, (**B**) cover of macroalgae, (**C**) cover of CCA and EAM, (**D**) density of juvenile corals, (**E**) total herbivorous fish biomass, (**F**) browsing fish biomass, and (**G**) grazing fish biomass among three habitats and five sites. Significant results (p<0.05) are given in bold.
(DOCX)

Table S3 **Summary of SNK multiple comparison tests to identify differences in benthic communities and herbivorous fish communities among sites and habitats on Lord Howe Island.** (**A**) Cover of live scleractinian coral, (**B**) cover of macroalgae, (**C**) density of juvenile corals, cover, (**D**) of CCA and EAM, (**E**) total herbivorous fish biomass, (**F**) browsing fish biomass, and (**G**) grazing fish biomass among three habitats and five sites. Significant results (p<0.05) are given in bold.
(DOCX)
