# Supplementary material for: High Macroalgal Cover and Low Coral Recruitment Undermines the Potential Resilience of the World's Southernmost Coral Reef Assemblages
Source: PLoS One. 2011 Oct 3;6(10):e25824. doi: 10.1371/journal.pone.0025824 (PMC3185058; doi:10.1371/journal.pone.0025824)
Supplement: Table S1 — Spatial variation in macroalgal assemblages of Lord Howe Island. Summary of the composition of macroalgal assemblages across three habitats within each of five sites on Lord Howe Island. Mean percentage cover of each of the major macroalgal taxa (based on six 50-m transects) are given. (DOCX) [file pone.0025824.s001.docx]

|  | Site 1 | | Site 2 | | | Site 3 | | | Site 4 | | | Site 5 | | |
| --- | --- | --- | --- | --- | --- | --- | --- | --- | --- | --- | --- | --- | --- | --- |
|  | Back | Slope | Back | Crest | Slope | Back | Crest | Slope | Back | Crest | Slope | Back | Crest | Slope |
| CHLOROPHYTA |  |  |  |  |  |  |  |  |  |  |  |  |  |  |
| *Caulerpa* | 1.0 | 3.7 | 2.7 | 9.3 | 7.7 | 2.7 | 3.0 | 5.3 |  | 2.7 | 5.7 | 2.3 | 42.7 | 30.7 |
| *Chlorodesmis* |  | 0.7 |  | 1.7 | 0.7 |  | 2.0 | 0.3 |  | 1.7 | 0.3 |  | 4.7 | 0.3 |
| *Codium* |  | 1.0 |  | 1.3 |  |  | 2.0 | 0.3 |  | 2.0 |  |  |  |  |
| *Neomeris* |  |  |  |  |  |  |  |  |  |  |  | 0.3 |  |  |
| Unident. fleshy green |  |  |  |  |  |  |  |  | 1.0 |  |  | 1.0 |  |  |
| RHODOPHYTA |  |  |  |  |  |  |  |  |  |  |  |  |  |  |
| *Asparagopsis* |  | 8.0 | 1.7 | 0.3 | 26.0 |  | 0.7 | 7.3 | 0.7 |  | 0.3 |  | 14.3 | 0.3 |
| *Acanthopohora* | 0.3 |  |  | 0.3 |  |  |  |  | 0.3 | 0.3 |  |  |  |  |
| *Laurencia brongniartii* |  |  |  | 0.7 |  |  | 0.7 | 0.3 |  |  |  |  | 0.3 |  |
| *Laurencia* sp. |  | 0.7 |  |  |  |  |  |  | 0.7 | 1.0 |  |  | 8.7 |  |
| *Plocamium hamatum* |  |  |  |  |  |  |  |  |  |  |  | 3.3 |  |  |
| Unident. foliose red |  |  |  |  |  |  |  |  |  | 0.7 | 1.3 |  |  |  |
| PHAEOPHYCEAE |  |  |  |  |  |  |  |  |  |  |  |  |  |  |
| *Dictyota* |  | 4.7 |  | 10.7 | 2.3 | 2.3 | 5.0 | 0.7 | 7.7 | 6.0 | 1.0 | 5.3 | 9.0 | 6.7 |
| *Lobophora* |  | 0.3 |  |  |  |  |  |  | 0.3 |  |  | 0.3 |  | 0.3 |
| *Padina* | 1.7 |  | 0.7 |  |  |  |  |  | 1.0 |  |  |  | 0.3 | 0.3 |
| *Sarcodia ciliata* |  |  |  |  |  |  |  |  |  |  |  |  | 6.0 |  |

**Table S1** **Spatial variation in macroalgal assemblages of Lord Howe Island**. Summary of the composition of macroalgal assemblages across three habitats within each of five sites on Lord Howe Island. Mean percentage cover of each of the major macroalgal taxa (based on six 50-m transects) are given.
