# Supplementary material for: High Macroalgal Cover and Low Coral Recruitment Undermines the Potential Resilience of the World's Southernmost Coral Reef Assemblages
Source: PLoS One. 2011 Oct 3;6(10):e25824. doi: 10.1371/journal.pone.0025824 (PMC3185058; doi:10.1371/journal.pone.0025824)
Supplement: Table S2 — Summary of 2-way ANOVA's comparing benthic and fish communities among habitats and sites on Lord Howe Island. Variation in (A) cover of live scleractinian coral, (B) cover of macroalgae, (C) cover of CCA and EAM, (D) density of juvenile corals, (E) total herbivorous fish biomass, (F) browsing fish biomass, and (G) grazing fish biomass among three habitats and five sites. Significant results (p<0.05) are given in bold. (DOCX) [file pone.0025824.s002.docx]

| Source of variation | SS | df | MS | F | p |
| --- | --- | --- | --- | --- | --- |
|  | | | | | |
| **(A) Live coral** | | | | | |
| **Habitat** | **0.904** | **2** | **0.452** | **17.605** | **< 0.001** |
| **Site** | **0.762** | **4** | **0.191** | **7.423** | **< 0.001** |
| **Habitat x Site** | **1.005** | **7** | **0.144** | **5.590** | **< 0.001** |
| Error | 1.797 | 70 | 0.026 |  |  |
|  |  |  |  |  |  |
| **(B) Macroalgal cover** | | | | | |
| **Habitat** | **2.429** | **2** | **1.214** | **56.919** | **< 0.001** |
| **Site** | **2.090** | **4** | **0.523** | **24.493** | **< 0.001** |
| **Habitat x Site** | **1.571** | **7** | **0.224** | **10.520** | **< 0.001** |
| Error | 1.493 | 70 | 0.021 |  |  |
|  |  |  |  |  |  |
| **(C) CCA and EAM** | | | | | |
| **Habitat** | **0.529** | **2** | **0.264** | **7.846** | **< 0.001** |
| **Site** | **1.008** | **4** | **0.252** | **7.477** | **< 0.001** |
| Habitat x Site | 0.342 | 7 | 0.049 | 1.450 | 0.199 |
| Error | 2.359 | 70 | 0.034 |  |  |
|  |  |  |  |  |  |
| **(D) Juvenile corals** | | | | | |
| **Habitat** | **0.767** | **2** | **0.384** | **5.858** | **0.004** |
| **Site** | **1.501** | **4** | **0.375** | **5.728** | **< 0.001** |
| **Habitat x Site** | **5.350** | **7** | **0.764** | **11.669** | **< 0.001** |
| Error | 4.585 | 70 | 0.066 |  |  |
|  |  |  |  |  |  |
| **(E) Total herbivorous fish biomass** | | | | | |
| **Habitat** | **201.381** | **2** | **100.691** | **17.091** | **< 0.001** |
| **Site** | **165.306** | **4** | **41.327** | **7.015** | **< 0.001** |
| Habitat x Site | 101.234 | 7 | 14.462 | 2.455 | 0.026 |
| Error | 412.393 | 70 | 5.891 |  |  |
|  |  |  |  |  |  |
| **(F) Browsing fish biomass** | | | | | |
| **Habitat** | **576.237** | **2** | **288.119** | **36.702** | **< 0.001** |
| **Site** | **257.565** | **4** | **64.391** | **8.203** | **< 0.001** |
| Habitat x Site | 103.710 | 7 | 14.816 | 1.887 | 0.085 |
| Error | 549.516 | 70 | 7.850 |  |  |
|  |  |  |  |  |  |
| **(G) Grazing fish biomass** | | | | | |
| Habitat | 16.487 | 2 | 8.244 | 1.584 | 0.212 |
| **Site** | **72.933** | **4** | **18.233** | **3.504** | **0.012** |
| Habitat x Site | 68.212 | 7 | 9.745 | 1.872 | 0.087 |
| Error | 364.249 | 70 | 5.204 |  |  |

**Table S2 Summary of 2-way ANOVA’s comparing benthic and fish communities among habitats and sites on Lord Howe Island.** Variation in (**A**) cover of live scleractinian coral, (**B**) cover of macroalgae, (**C**) cover of CCA and EAM, (**D**) density of juvenile corals, (**E**) total herbivorous fish biomass**,** (**F**) browsing fish biomass, and (**G**) grazing fish biomass among three habitats and five sites. Bonferroni correction was used to account for the multiple comparisons among herbivorous fish groups (α = 0.017). Significant results are given in bold.
