# Supplementary material for: High Macroalgal Cover and Low Coral Recruitment Undermines the Potential Resilience of the World's Southernmost Coral Reef Assemblages
Source: PLoS One. 2011 Oct 3;6(10):e25824. doi: 10.1371/journal.pone.0025824 (PMC3185058; doi:10.1371/journal.pone.0025824)
Supplement: Table S3 — Summary of SNK multiple comparison tests to identify differences in benthic communities and herbivorous fish communities among sites and habitats on Lord Howe Island. (A) Cover of live scleractinian coral, (B) cover of macroalgae, (C) density of juvenile corals, cover, (D) of CCA and EAM, (E) total herbivorous fish biomass, (F) browsing fish biomass, and (G) grazing fish biomass among three habitats and five sites. Significant results (p<0.05) are given in bold. (DOCX) [file pone.0025824.s003.docx]

|  | S1Back | S1Slope | S2Back | S2Slope | S2Crest | S3Back | S3Slope | S3Crest | S4Back | S4Slope | S4Crest | S5Back | S5Slope |
| --- | --- | --- | --- | --- | --- | --- | --- | --- | --- | --- | --- | --- | --- |
| S1 Back |  |  |  |  |  |  |  |  |  |  |  |  |  |
| S1 Slope | 0.387 |  |  |  |  |  |  |  |  |  |  |  |  |
| S2 Back | 0.169 | 0.977 |  |  |  |  |  |  |  |  |  |  |  |
| S2 Slope | 0.541 | 0.998 | 0.999 |  |  |  |  |  |  |  |  |  |  |
| S2 Crest | 0.467 | 0.997 | 0.996 | 0.851 |  |  |  |  |  |  |  |  |  |
| S3 Back | 0.680 | 0.272 | 0.176 | 0.327 | 0.262 |  |  |  |  |  |  |  |  |
| S3 Slope | 0.481 | 0.970 | 0.995 | 0.996 | 0.993 | 0.327 |  |  |  |  |  |  |  |
| S3 Crest | 0.490 | 0.994 | 0.996 | 0.989 | 0.978 | 0.294 | 0.983 |  |  |  |  |  |  |
| S4 Back | 0.225 | 0.963 | 0.954 | 0.881 | 0.847 | 0.100 | 0.951 | 0.943 |  |  |  |  |  |
| S4 Slope | 0.440 | 0.982 | 0.992 | 0.999 | 0.996 | 0.264 | 0.938 | 0.981 | 0.971 |  |  |  |  |
| S4 Crest | 0.259 | 0.990 | 0.846 | 1.000 | 0.999 | 0.199 | 0.999 | 0.998 | 0.975 | 0.995 |  |  |  |
| S5 Back | 0.401 | 0.945 | 0.984 | 1.000 | 0.999 | 0.245 | 0.779 | 0.996 | 0.981 | 0.954 | 0.987 |  |  |
| S5 Slope | 0.480 | 0.998 | 0.997 | 0.970 | 0.962 | 0.267 | 0.996 | 0.995 | 0.617 | 0.999 | 0.999 | 1.000 |  |
| S5 Crest | **< 0.001** | **< 0.001** | **< 0.001** | **< 0.001** | **< 0.001** | **< 0.001** | **< 0.001** | **< 0.001** | **< 0.001** | **< 0.001** | **< 0.001** | **< 0.001** | **< 0.001** |

**Table S3 Summary of SNK multiple comparison tests to identify differences in benthic communities and herbivorous fish communities among sites and habitats on Lord Howe Island.** (**A**) Cover of live scleractinian coral, (**B**) cover of macroalgae, (**C**) density of juvenile corals, cover, (**D**) of CCA and EAM, (**E**) total herbivorous fish biomass**,** (**F**) browsing fish biomass, and (**G**) grazing fish biomass among three habitats and five sites. Significant results (p < 0.05) are given in bold.

**A**: Live scleractinian coral. Summary of SNK multiple comparison tests to identify differences in the cover of live scleractinian coral among sites and habitats on Lord Howe Island.

**B**: Fleshy macroalgae. Summary of SNK multiple comparison tests to identify differences in the cover of fleshy macroalgae among sites and habitats on Lord Howe Island.

|  | S1Back | S1Slope | S2Back | S2Slope | S2Crest | S3Back | S3Slope | S3Crest | S4Back | S4Slope | S4Crest | S5Back | S5Slope |
| --- | --- | --- | --- | --- | --- | --- | --- | --- | --- | --- | --- | --- | --- |
| S1 Back |  |  |  |  |  |  |  |  |  |  |  |  |  |
| S1 Slope | **0.009** |  |  |  |  |  |  |  |  |  |  |  |  |
| S2 Back | 0.548 | **0.044** |  |  |  |  |  |  |  |  |  |  |  |
| S2 Slope | **< 0.001** | 0.074 | **< 0.001** |  |  |  |  |  |  |  |  |  |  |
| S2 Crest | **0.001** | 0.434 | **0.005** | 0.154 |  |  |  |  |  |  |  |  |  |
| S3 Back | 0.796 | **0.040** | 0.968 | **< 0.001** | **0.005** |  |  |  |  |  |  |  |  |
| S3 Slope | 0.068 | 0.784 | 0.204 | **0.027** | 0.425 | 0.167 |  |  |  |  |  |  |  |
| S3 Crest | 0.084 | 0.620 | 0.252 | **0.019** | 0.320 | 0.219 | 0.998 |  |  |  |  |  |  |
| S4 Back | 0.122 | 0.695 | 0.299 | **0.013** | 0.308 | 0.232 | 0.722 | 0.931 |  |  |  |  |  |
| S4 Slope | 0.204 | 0.507 | 0.357 | **0.003** | 0.154 | 0.185 | 0.788 | 0.883 | 0.834 |  |  |  |  |
| S4 Crest | 0.062 | 0.464 | 0.207 | **0.021** | 0.286 | 0.185 | 0.978 | 0.843 | 0.944 | 0.866 |  |  |  |
| S5 Back | 0.114 | 0.725 | 0.261 | **0.012** | 0.314 | 0.175 | 0.888 | 0.966 | 0.914 | 0.643 | 0.963 |  |  |
| S5 Slope | **< 0.001** | **0.048** | **< 0.001** | 0.678 | 0.159 | **< 0.001** | **0.011** | **0.008** | **0.005** | **0.001** | **0.010** | **0.004** |  |
| S5 Crest | **< 0.001** | **< 0.001** | **< 0.001** | **< 0.001** | **< 0.001** | **< 0.001** | **< 0.001** | **< 0.001** | **< 0.001** | **< 0.001** | **< 0.001** | **< 0.001** | **< 0.001** |

**C**: Juvenile corals. Summary of SNK multiple comparison tests to identify differences in the density of juvenile corals among sites and habitats on Lord Howe Island.

|  | S1Back | S1Slope | S2Back | S2Slope | S2Crest | S3Back | S3Slope | S3Crest | S4Back | S4Slope | S4Crest | S5Back | S5Slope |
| --- | --- | --- | --- | --- | --- | --- | --- | --- | --- | --- | --- | --- | --- |
| S1 Back |  |  |  |  |  |  |  |  |  |  |  |  |  |
| S1 Slope | 0.485 |  |  |  |  |  |  |  |  |  |  |  |  |
| S2 Back | 0.293 | **0.018** |  |  |  |  |  |  |  |  |  |  |  |
| S2 Slope | 0.176 | **0.037** | 0.911 |  |  |  |  |  |  |  |  |  |  |
| S2 Crest | 0.256 | 0.601 | **0.004** | **0.011** |  |  |  |  |  |  |  |  |  |
| S3 Back | **0.026** | **< 0.001** | 0.345 | 0.374 | **< 0.001** |  |  |  |  |  |  |  |  |
| S3 Slope | 0.478 | 0.939 | **0.031** | **0.049** | 0.824 | **0.001** |  |  |  |  |  |  |  |
| S3 Crest | 0.143 | 0.637 | **0.002** | **0.004** | 0.703 | **< 0.001** | 0.724 |  |  |  |  |  |  |
| S4 Back | 0.276 | **0.029** | 0.819 | 0.857 | **0.008** | 0.368 | **0.043** | **0.003** |  |  |  |  |  |
| S4 Slope | 0.460 | 0.911 | **0.021** | **0.038** | 0.800 | **< 0.001** | 0.822 | 0.738 | **0.031** |  |  |  |  |
| S4 Crest | 0.799 | 0.665 | 0.165 | 0.196 | 0.461 | **0.007** | 0.424 | 0.325 | 0.197 | 0.561 |  |  |  |
| S5 Back | 0.534 | 0.777 | 0.127 | 0.122 | 0.549 | **0.005** | 0.694 | 0.386 | 0.141 | 0.725 | 0.990 |  |  |
| S5 Slope | 0.102 | **0.002** | 0.472 | 0.670 | **0.001** | 0.499 | **0.005** | **< 0.001** | 0.609 | **0.003** | **0.038** | **0.030** |  |
| S5 Crest | **0.001** | **< 0.001** | 0.052 | **0.039** | **< 0.001** | 0.228 | **< 0.001** | **< 0.001** | **0.044** | **< 0.001** | **< 0.001** | **< 0.001** | 0.147 |

**D**: EAM and CCA. Summary of SNK multiple comparison tests to identify differences in the cover of EAM and CCA among sites and habitats on Lord Howe Island.

| **Sites** | | | | |
| --- | --- | --- | --- | --- |
|  | Site1 | Site2 | Site3 | Site4 |
| Site1 |  |  |  |  |
| Site2 | 0.830 |  |  |  |
| Site3 | 0.364 | 0.499 |  |  |
| Site4 | 0.176 | 0.191 | 0.377 |  |
| Site5 | **0.008** | **0.006** | **0.001** | **< 0.001** |
|  |  |  |  |  |
| **Habitats** | | | | |
|  | Back | Slope |  |  |
| Back |  |  |  |  |
| Slope | **0.001** |  |  |  |
| Crest | **0.002** | 0.970 |  |  |

**E**: Herbivorous fishes. Summary of SNK multiple comparison tests to identify differences in the biomass of all herbivorous fishes among sites and habitats on Lord Howe Island.

| **Sites** | | | | |
| --- | --- | --- | --- | --- |
|  | Site1 | Site2 | Site3 | Site4 |
| Site1 |  |  |  |  |
| Site2 | 0.756 |  |  |  |
| Site3 | **0.014** | **0.017** |  |  |
| Site4 | 0.191 | 0.210 | **< 0.001** |  |
| Site5 | 0.252 | 0.201 | **0.001** | 0.677 |
|  |  |  |  |  |
| **Habitats** | | | | |
|  | Back | Slope |  |  |
| Back |  |  |  |  |
| Slope | **< 0.001** |  |  |  |
| Crest | 0.116 | **< 0.001** |  |  |

**F**: Macroalgal browsing fishes. Summary of SNK multiple comparison tests to identify differences in the biomass of macroalgal browsing fishes among sites and habitats on Lord Howe Island

| **Sites** | | | | |
| --- | --- | --- | --- | --- |
|  | Site1 | Site2 | Site3 | Site4 |
| Site1 |  |  |  |  |
| Site2 | 0.972 |  |  |  |
| Site3 | **0.001** | **0.001** |  |  |
| Site4 | 0.205 | 0.391 | **< 0.001** |  |
| Site5 | 0.746 | 0.489 | **0.002** | 0.193 |
|  |  |  |  |  |
| **Habitats** | | | | |
|  | Back | Slope |  |  |
| Back |  |  |  |  |
| Slope | **< 0.001** |  |  |  |
| Crest | 0.056 | **< 0.001** |  |  |

**G**: Grazing fishes. Summary of SNK multiple comparison tests to identify differences in the biomass of grazing fishes among sites on Lord Howe Island.

|  | Site1 | Site2 | Site3 | Site4 |
| --- | --- | --- | --- | --- |
| Site1 |  |  |  |  |
| Site2 | 0.762 |  |  |  |
| Site3 | 0.320 | 0.255 |  |  |
| Site4 | 0.331 | 0.348 | 0.804 |  |
| Site5 | **0.008** | **0.012** | 0.115 | 0.081 |
